# Physicochemical characterization and *in vitro* evaluation of the antioxidant and anticandidal activities of Moroccan propolis

**DOI:** 10.14202/vetworld.2022.341-349

**Published:** 2022-02-15

**Authors:** Abderrazak Aboulghazi, Soumaya Touzani, Mouhcine Fadil, Badiaa Lyoussi

**Affiliations:** 1 Department of Biology, Laboratory of Natural Substances, Pharmacology, Environment, Modeling, Health, and Quality of Life (SNAMOPEQ), Faculty of Sciences Dhar Mehraz, Sidi Mohamed Ben Abdellah University, Fez 30000, Morocco; 2 Physicochemical Laboratory of Inorganic and Organic Materials, Materials Science Center, Mohammed V University in Rabat, Morocco

**Keywords:** antioxidant activity, propolis, total flavonoids, total polyphenols, vulvovaginal candidiasis

## Abstract

**Background and Aim:**

Human mycotic infections are one of the major health problems worldwide. Prolonged use of antimycotic drugs has contributed to the development of resistance in pathogenic fungi. This study was conducted to examine antioxidant and anticandidal activities of Moroccan propolis.

**Materials and Methods:**

Two ethanolic extracts of Moroccan propolis from the Fez-Meknes region were evaluated regarding the following physicochemical parameters: Yield, pH, total carbohydrates, total proteins, total lipids, minerals, total phenolic content, total flavonoid content, and antioxidant activity using ferric reducing antioxidant power (FRAP) and 2,2’-azino-bis (3-ethylbenzothiazoline-6-sulfonic acid) (ABTS) assays. In addition, we assessed the *in vitro* anticandidal activity against vulvovaginal candidiasis strains, that is, *Candida albicans*, *Candida glabrata*, *Candida parapsilosis*, and *Candida krusei*, using the broth micromethod according to the CLSI/M27-A3 reference guidelines.

**Results:**

The propolis samples exhibited a mean yield of 16%, with an acidic pH ranging from 4.8 to 5.9; the sample from the Oued Amlil area (OAPEE) contained high levels of resin, balsam, moisture, total carbohydrates, and total lipids: 59.8%, 0.71%, 2%, 1.01 gGlcEq/g, and 120 mg/g, respectively. Moreover, the sample from the Sefrou area (SFPEE) was richer in total proteins and minerals, with values of 2.5 g/100 g and 1.84%, respectively. The total polyphenol and flavonoid content in the propolis extracts were 117.38 and 194.68 mg of gallic acid equivalent/g, and 17.45–27.79 mg of quercetin equivalent/g, respectively. Regarding the antioxidant activity, the most effective propolis extract was the sample from the Sefrou area, at 72.5 μg/mL and 118.78 μmoL Fe^2+^/g for ABTS-half-maximal inhibitory concentration and FRAP-half maximal effective concentration, respectively. The analysis of phenolic compounds using high-performance liquid chromatography with a diode-array detector revealed the presence of 13 polyphenols. The main compound in the OAPEE sample was epicatechin (310 mg/g), whereas in the SFPEE sample was apigenin (410 mg/g). Regarding the antifungal activity against *Candida* species, the minimum inhibitory concentration and minimum fungicidal concentration of the Moroccan propolis ethanolic extracts ranged between 31.2 and 62.5 μg/mL and 62.5 and 125 μg/mL, respectively, comparable with fluconazole (as a reference antimycotic).

**Conclusion:**

This study suggests that Moroccan propolis (31.2 and 125 μg/mL) may be an important source of bioactive molecules with anticandidal activity. Propolis may be a promising naturally-occurring candidate for the development of antimycotic drugs.

## Introduction

About 75% of women with sexual activity have at least one episode of mycotic infection over their lifespan [1]. Mycotic infections are the most common reason for gynecology consultation [2], are caused by the commensal and saprophytic yeast *Candida*. This microorganism may, in some situations, become a pathogen causing local infections, that is, vulvovaginal candidiasis [3]. Although *Candida albicans* remains the species that are isolated most often during these infections, a considerable increase in the frequency of non-*C. albicans* species has also been highlighted, mainly *Candida glabrata*, which is isolated in 5-15% of cases of vulvovaginal candidiasis, as well as *Candida parapsilosis*, *Candida tropicalis*, and *Candida krusei* [4]. In recent years, antifungal resistance has become an increasing problem associated with the fungus *Candida*, and the optimization of therapies for candidiasis has been the focus of broad research. Moreover, the antifungal drugs available for the treatment of candidiasis infections are quite restricted, being limited to polyenic and azolic chemicals. Fluconazole is one of the most common agents used to treat vulvovaginal candidiasis [5].

These various difficulties have stimulated our interest in researching other effective, safe, and low-cost alternative substances, from beehive products to synthetic antifungal drugs. In Morocco, as in many other countries, beehive products are used in traditional therapy, despite the discovery of pharmaceutical synthetic processes [6]. They are considered a real treasure of bioactive compounds and an essential raw material for developing new drugs [7]. One of these products is propolis, a strongly resinous mixture of various amounts of beeswax produced by bees by mixing their saliva with resins collected from plants, particularly from flowers and leaf buds [8]. Propolis exhibits important anti-inflammatory, antimicrobial, and immunostimulatory activities [9,10]. In addition, it contains a variety of chemical compounds, such as phenolic acids and flavonoids. The chemical composition of propolis is strongly dependent on the area from which it is collected [11].

Despite the anticandidal activity of propolis, only a few studies have been carried out to determine the inhibitory effect of Moroccan propolis samples against candidiasis-causing pathogens [12,13]. Therefore, this study aimed to determine the physicochemical properties, antioxidant activity, and phenolic profile using high-performance liquid chromatography with diode array detection (HPLC-DAD), as well as to investigate the anticandidal activity of Moroccan propolis.

## Materials and Methods

### Ethical approval

The study was carried out according to the ethical approval obtained from Sidi Mohamed Ben Abdallah University, Fez, Morocco, under the responsibility of the Laboratory of Natural Substances, Pharmacology, Environment, Modeling, Health and Quality of Life (SNAMOPEQ), Faculty of Sciences, Dhar El Mahraz (L.20.USMBA-SNAMOPEQ).

### Study period and location

The study was carried out from November 2019 to January 2020 at Laboratory of Natural Substances, Pharmacology, Environment, Modeling, Health and Quality of Life, Faculty of Sciences Dhar El Mahraz.

### Chemicals and reagents

Folin-Ciocalteu reagent, sulfuric acid (H_2_SO_4_), potassium-ferricyanide, 2,2-azino-bis (3-ethyl-benzothiazoline-6-sulphonic acid) (ABTS), sodium nitrite, sodium hydroxide, sodium carbonate, trichloroacetic acid, trisodium phosphate, potassium dihydrogen phosphate, and dipotassium hydrogen phosphate anhydrous, ascorbic acid, dimethyl sulfoxide (DMSO1%), phosphate-buffered solution, bovine serum albumin (BSA), acetate buffer (pH=3.6), (2,4,6-tripyridyl-s-triazine) (TPTZ), and hydrochloric acid (HCl) were procured from Merck industry (Germany). Ferric-chloride hexahydrate (FeCl_3_-6H_2_O), H_2_SO_4_, anthrone, vanillin, aluminum trichloride (AlCl_3_), 2,3,5-triphenyl tetrazolium chloride (TTC) Sabouraud Dextrose agar, and broth culture medium (SDA, SDB) were obtained from Sigma-Aldrich Chemical Co. (United States of America,). Fluconazole (Diflucan 150 mg) was obtained from Pfizer (Morocco), vanillic acid, epicatechin, coumaric acid, ferulic acid, chlorogenic acid, ellagic acid, hesperidin, cinnamic acid, rutin, apigenin, quercetin, rosmarinic acid, naringin, and kaempferol was purchased from Abcam, United Kingdom. All chemicals were of analytical grade.

### Propolis samples

After honey extraction, two propolis samples were directly collected by beekeepers from *Apis mellifera* hives located in the Fez-Meknes region (Table-1) [11], by scratching the hive walls and frames, followed by the removal of debris of wood and bees. The samples were protected from light and immediately transferred to the laboratory in a plastic food bag at – 20°C.

**Table 1:** Harvest date, color, and botanical origins of Moroccan propolis samples.

Sample	Area	Geolocalisation	Vegetation Origin	Collection date	Color
OAPEE	Oued Amlil	34°12’0”N4°18’48”W	Mixed native: *Olea, Eucalyptus, Orange, Silybum*.	December 2018	Brown
SFPEE	Sefrou	33°49’48”N4°54’58”W	Mixed native: *Olea, Pinus, Juniperus, Rosmarinus, Cistus, Lavandula*, and *Pistacia* [11]	December 2019	Dark brown

OAPEE=Oued Amlil propolis ethanolic extrac, SFPEE=Sefrou propolis ethanolic extract

### *Candida* strains

All clinical yeast strains used here were isolated from cases of vulvovaginal candidiasis and subsequently identified according to the standardized protocols of the University Hospital Center of Fez and to the classical mycological tests for *Candida* species, such as germ tube formation on fresh Human serum at 35°C, microscopic morphology, and growth at 35°C for 24-48 h.

Four *Candida* species were identified: *C. albicans*, *C. glabrata*, *C. parapsilosis*, and *C. krusei*. Strains were subcultured on SDA culture medium at 35°C for 24 h to ensure the purity and viability of the inocula.

### Extraction of balsam and moisture content

Propolis was frozen at −80°C and then pounded in a mortar until a uniform particles size was obtained. Three grams of each sample were dissolved in 30 mL of 70% hydro-ethanolic solution (70:30 ethanol: water), and the resulting mixture was stirred constantly for 3 h in a dark room. An ethanol/water mixture (70/30) is the most common solvent used for propolis extraction, as it is non-toxic and efficient, particularly for polyphenol and flavonoid extraction [14]. Subsequently, the propolis ethanolic extract was separated by a 5 min centrifugation at 2800 x g, and the supernatant was filtrated on Whatman paper, Grade 3, as described by Popova *et al*. [15]. The supernatant was collected in a volumetric flask and completed up to 100 mL using the same 70% ethanol solvent. The final filtrates represent the balsam of propolis and are referred as propolis ethanolic extract (PEE). The yield was expressed as balsam content (soluble ethanolic fraction) and determined according to Bankova *et al*. [16]. Fifty milliliters of each ethanolic extract were evaporated to dryness on a rotary evaporator under reduced pressure at 40°C. The moisture content was determined as a percentage weighting of 1 g of propolis that was oven-dried at 40°C for16 h.

### Wax, resin, and total ash level determination

The wax and resin levels in the propolis samples studied here were determined according to the method described by Papotti *et al*. [17], with some modifications. Briefly, 1 g of propolis was added to 40 mL of ether petroleum and heated to 40-60°C under magnetic stirring for 48 h. Subsequently, 40 mL of 70% ethanol was added to the heated mixture under reflux until a clear solution was obtained, and the mixture was then cooled at 0°C for 1 h, to promote wax separation. The results were expressed as a percentage (w/w), representing the rate of wax in the propolis sample. To determine the total ash content, an analysis was performed according to the Association of Official Agricultural Chemists [18].

### Preparation of dilutions

Serial dilutions of the ethanolic extract of propolis and fluconazole standard were prepared in 1% DMSO using the following ranges from 19 to 500 μg/mL and from 20 to 150 μg/mL, respectively.

### Yield of the extraction procedure

The yield of the extraction procedure was evaluated by comparing the dry weight of the extract with the initial weight of propolis used in the extraction using the formula:


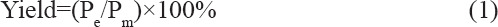


Where P_e_ is the weight of propolis extract (g) and P_m_ is the weight of raw propolis (g).

### pH determination

To determine the pH of the propolis samples, a digital pH meter (model 2005, J. P. Selecta, Spain) was used. The selective ion electrode and reference electrode were placed on the support holder with 5 mL of PEE [19]. The pH values were registered after calibration of the pH meter readings using three buffer solutions: pH 4.0, pH 6.86, and pH 9.18.

### Determination of primary metabolites

#### Total carbohydrates

The aliquots of PEE were dissolved in 1 mL of distilled water, followed by the addition of 4 mL of anthrone reagent prepared in H_2_SO_4_. The solution was incubated for 10 min in boiling water and the absorbance was measured at 630 nm. A standard curve was prepared using glucose [20].

#### Total proteins

The total protein content of the propolis extracts was estimated using Lowry’s method [21] using bovine serum albumin as a standard. The final results are expressed as mg of BSA equivalent per 100 g of propolis.

#### Total lipids

The total lipid content of the propolis extracts was determined according to the single-step method using olive oil as the standard [22]. One milliliter of PEE was mixed with 1.5 mL of concentrated H_2_SO_4_ incubated in a water bath for 10 min. After cooling, 2.4 mL of vanillin reagent was added, the mixture was incubated for 40 min at room temperature, and the absorbance was measured at 490 nm.

### Mineral composition

The mineral composition of the propolis extracts (sodium, potassium, calcium, and magnesium) was determined using inductively coupled plasma mass spectrometry. Initially, 5 mL of Nitric acid (HNO_3_) was added to 0.2 g of mineralized propolis and a power of 1000 W was applied for 5 min. Subsequently, 5 mL of HNO_3_ and 1 mL of 30% Hydrogen peroxide were added to the solution. All samples were cooled to room temperature, made up to 100 mL with distilled water, and stored at 4°C until analysis [23].

### Determination of secondary metabolites

#### Total phenolic and flavonoid content

The total polyphenol and flavonoid contents were determined using the Folin-Ciocalteu reagent and AlCl_3_ method, as described by Galeotti *et al*. [24]. The results were expressed as mg of gallic acid equivalent per gram of propolis (mg GAE/g) for total polyphenol content and mg of quercetin equivalent per gram of propolis (mg QE/g) for total flavonoid content. To avoid the overestimation of flavonoid content in propolis, a color correction was carried out by preparing a blank under the same experimental conditions using the same amount of propolis and replacing the volume of reagents used in the test with distilled water. The absorbance of this mixture was measured and then subtracted from the initial absorbance obtained in the test.

### ABTS assay

Briefly, 2.5 mL of ABTS reagent was mixed with 50 μL of sample and incubated at room temperature for 6 min. After incubation, the absorbance was measured at 734 nm using 100% methanol as a control [25]. The ABTS scavenging activity was calculated using the following formula:


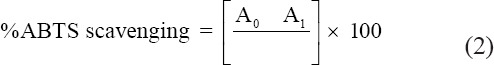


Where A_0_ and A_1_ are the absorbances of the control and the sample, respectively, ascorbic acid was used as a positive control and tests were carried out in duplicate.

### Ferric reducing antioxidant power (FRAP) assay

The FRAP working solution was freshly prepared each time: 0.3 M acetate buffer (pH=3.6), 0.01 M TPTZ in 0.04 M HCl, and 0.02M FeCl_3_ 6H_2_O were mixed at 10:1:1 (v/v/v) and kept away from light. Then, 0.075 mL of PEE were added to 2.25 mL of FRAP working solution and 0.225 mL of deionized water, and the mixture was vortexed and incubated at 37°C for 30 min. A calibration curve was prepared using ferrous sulfate (200, 400, 600, 80, and 1000 μM). Absorbance was recorded at 593 nm and the results were expressed as μmoL Fe^2+^/g [26]. Quercetin was used as a positive control and tests were carried out in duplicate.

### HPLC-DAD analysis

The dried ethanolic extract of each propolis sample was diluted, added to methanol, and filtered through a 0.45 mM membrane filter syringe before injection onto a Shimadzu prominence system equipped with a diode-array detector, a degasser, a quaternary pump (LC A20), and a Ryodine type injector. Polyphenol separation was carried out on a C18 column (Agilent Zorbax; dimensions: 4.6 mm×250 mm×5 μM). The flow rate was 1 mL/min of a mobile phase composed of a ternary gradient of acetonitrile, methanol, and acidified water; the temperature of the column was 30°C; and the injection volume was 20 μL. Under the same conditions, standard solutions, syringic acid, and tyrosol were injected, to determine the response factor. Pure compounds were used as standards, including:

- Phenolic acids: Vanillic acid, coumaric acid, ferulic acid, cinnamic acid, gallic acid, chlorogenic acid, rosmarinic acid, and ellagic acid.
- Flavonoids: Hesperidin, epicatechin, rutin, apigenin, quercetin, naringin, and kaempferol.

Phenolic compounds were identified by comparing their ultraviolet-visible spectra and retention times with those of the corresponding standards, and chromatographic data were acquired using the LabSolutions software equipped with a spectral identification module for the separated compounds; the results are expressed as mg/g of propolis [27].

### *In vitro* antifungal susceptibility test

The antifungal activity was determined using microtitration plates and the TTC dye method for all *Candida* strains [28]. Briefly, inocula were prepared in 0.9% sterile saline, and their turbidity was adjusted to 0.5 McFarland. Initially, 170 μL of SDB medium was distributed in the plate wells, and 10 μL of each propolis ethanolic extract sample was transferred to the wells at concentrations ranging from 1.95 to 1000 μg/mL. Finally, 20 μL of adjusted inocula from each strain was added to each well. Positive and negative controls were also included (with and without *Candida* suspension, respectively). A serial dilution of fluconazole was used as a standard drug. The MIC values were determined after 24 h of incubation at 35°C; the lowest concentration that could visibly inhibit fungal growth was considered as the MIC. Subsequently, 10 μL of the TTC dye was used to confirm the presence of viable microorganisms, as it reflects the activity of the dehydrogenase enzymes involved in the process of cell respiration [29]. This technique is widely used to determine the MIC, because in the presence of bacteria or fungi, TTC is reduced to red-colored formazan, which is directly proportional to the quantity of viable cells [30]. This test was performed in duplicate.

After MIC determination, the content of the well corresponding to the MIC and the content of the two preceding concentrations were subcultured in SDA Petri dishes and incubated at 35°C for 48 h. The MFC was defined as the lowest concentration that was able to inhibit *Candida* species growth. The MFC/MIC ratio was calculated to determine whether the PEE had a fungistatic (MFC/MIC≥4) or fungicidal (MFC/MIC<4) activity [31].

### Statistical analysis

The tests were performed in duplicate, and the results were expressed as the mean±standard deviation. Statistical comparisons were carried out with a one-way analysis of variance using the Minitab 18 software (Minitab, Ltd., Brandon Court, Unit E1-E2 Progress Way, Coventry, CV3 2TE, UK).

## Results and Discussion

The physicochemical characterization of the Moroccan propolis samples studied here is presented in Table-2. The results showed that the extraction yields were significantly higher in the Sefrou propolis ethanolic extract (SFPEE) (18.3%) versus the Oued amlil propolis ethanolic extract (OAPEE) (15.8%) sample, and that both samples had an acidic pH. These results were similar to those obtained for Indonesian propolis, which exhibited a yield and pH of 18.3% and 5.4, respectively [32].

**Table 2:** Quantitative estimation of propolis physicochemical parameters.

Propolis Sample	Yield (%)	pH	Resin (%)	Wax (%)	Balsams (%)	Moisture (%)	Ash (%)
OAPEE	15.8±0.40	5.9±0.30*	59.8±2.20*	28.9±0.20*	0.71±0.05*	2.00±0.09*	1.79±0.19
SFPEE	18.3±1.80*	4.8±0.30	48.3±1.80	21.8±0.50	0.68±0.03	1.09±0.05	1.84±0.11*

SD=Standard deviation for duplicate determination; the results are presented as mean±SD

* (p<0.05).

OAPEE=Oued Amlil propolis ethanolic extract; SFPEE=Sefrou propolis ethanolic extract, SD=Standard deviation

In general, propolis consists of about 50% resin and 30% wax [33]. Our results indicated that the contents of resin and wax of the OAPEE sample were significantly higher than those of the SFPEE sample. In turn, balsam and moisture levels are used to determine propolis quality: A high humidity content in propolis indicates bad storage and manipulation conditions [34]. The studied propolis samples showed that the moisture and balsam levels in the OAPEE sample were significantly higher than those of the SFPEE sample (2.00%±0.09% vs. 1.09%±0.05%; and 0.71%±0.05% vs. 0.68%±0.03%), respectively. These results are within the ranges reported for Slovenian propolis samples [35].

The determination of total ash content is particularly important for propolis samples, as this analysis can identify the possible adulteration of the material through the presence of impurities, or even residues, from previously extracted propolis [36]. In the present study, the obtained ash values (1.79%±0.19% in the OAPEE and 1.84%±0.11% in the SFPEE sample) were similar to those obtained in propolis samples from Brazil [37].

The total carbohydrate content was similar in both samples (1.01±0.05 gGlcEq/g in the OAPEE vs. 0.98±0.03 gGlcEq/g in the SFPEE sample). In contrast, the content of total proteins was significantly higher in the OAPEE versus the SFPEE sample, with the opposite relationship being observed for total lipids (Table-3). The values obtained were similar to those detected in Iranian propolis [38]. In addition, minerals are among the essential micronutrients that exist in propolis and may contribute to the pharmacological properties of this beehive product [39]. A high content of minerals (sodium, potassium, calcium, and magnesium) was observed in the SFPEE sample. The values obtained in this study were slightly higher than those found for Malaysian propolis [40].

**Table 3:** Total carbohydrates, proteins, lipids, and minerals content in propolis samples.

Propolis Sample	T. carbs (gGlcEq/g)	T. prot (g/100g)	T. lip (mg/g)	Magnesium (µg/g)	Sodium (µg/g)	Potassium (µg/g)	Calcium (µg/g)
OAPEE	1.01±0.05	0.99±0.05	120±0.08*	6.9±0.02	18.6±0.03	25.7±0.02	6.4±0.02
SFPEE	0.98±0.03	2.5±0.09*	108±0.05	13.6±0.02**	33±0.02**	53.6±0.02**	10.9±0.02*

OAPEE=Oued Amlil propolis ethanolic extract, SFPEE=Sefrou propolis ethanolic extract. T. carbs: Total carbohydrates, T. Prot: Total proteins, T. Lip: Total lipids.

* SD=Standard deviation for duplicate determination; the results are presented as mean±SD (*p<0.05),

** (p<0.01)

The total polyphenol contents of the Moroccan propolis samples depended significantly on the sampling area and the surrounding flora of the hives (Table-1). The TPC level in the SFPEE sample was significantly higher than that of the OAPEE sample (194.68±2.07 mgGAE/g vs. 117.38±1.86 mgGAE/g) (Table-4). These values agree with those obtained for Algerian and Lithuanian propolis, in which the TPC ranged from 19.51 to 219.66 mgGAE/g and from 95.02 to 196.81 mgGAE/g [41,42], respectively. However, the TPC levels detected in this study were considerably higher than those found in propolis from Tunisia, which ranged from 17.34 to 33.4 mgGAE/g [43].

**Table 4:** Antioxidant activity of Moroccan propolis studied.

Sample	TPC (mg GAE/g)	TFC (mg QE/g)	FRAP-EC_50_ (µmoL Fe^2^+/g)	ABTS-IC_50_ (µg/mL)
OAPEE	117.38±1.86	17.45±1.06	100.57±2.13	115.5±0.67**
SFPEE	194.68±2.07*	24.79±0.78**	118.78±4.27*	72.50±0.42
BHT	-	-	-	-
Q	-	-	93.09±1.68	-
AA	-	-	-	28.6±1.09

OAPEE=Oued Amlil propolis ethanolic extract, SFPEE=Sefrou propolis ethanolic extract. TPC=Total polyphenols content, TFC=Total flavonoids content, Q=quercetin, AA=ascorbic acid.

* SD-standard deviation for duplicate determination; the results are presented as mean±SD (*p<0.05),

** (p<0.01). EC_50_=Half maximal effective concentration

The total flavonoid content of the studied propolis samples was 17.45±1.06 mgQE/g for the OAPEE and 24.79±0.78 mgQE/g for the SFPEE (Table-4). These values are similar to those obtained for a propolis extract from South Korea, in which TFC was present in the range of 21-50 mgQE/g [44], and lower than those obtained for propolis from Mexico (13-379 mgQE/g [45] and Turkey (522.71 mg QE/g) [46]. Whereas the obtained results were higher than those described for the propolis from Algeria (0.57-3.53 mgQE/g) [47]. These variations in the TPC and TFC values may be attributed to the botanical and geographic origins of the samples and bee species, the harvest year, and seasonal variations [48].

Two different methods were used to assess the antioxidant activity of propolis (ABTS and FRAP). Both samples possessed potent antioxidant activity, although with significant differences. The FRAP test showed that the half-maximal effective concentration of the OAPEE was 100.57±2.13 μmoL Fe^2+^/g, whereas that of the SFPEE was 118.78±4.27 μmoL Fe^2+^/g. Conversely, the Half-maximal inhibitory concentration (IC_50_) values of the ABTS test revealed that the SFPEE (IC_50_=72.50±0.42 μg/mL) was more potent than the OAPEE (IC_50_=115.5±0.67) (Table-4). The results of the ABTS test were in the range of those obtained for Palestinian propolis [49] and higher than those found in Greek propolis [50]. For the FRAP assay, the obtained values were higher than those obtained for propolis from Venezuela [51].

**Figure-1:**
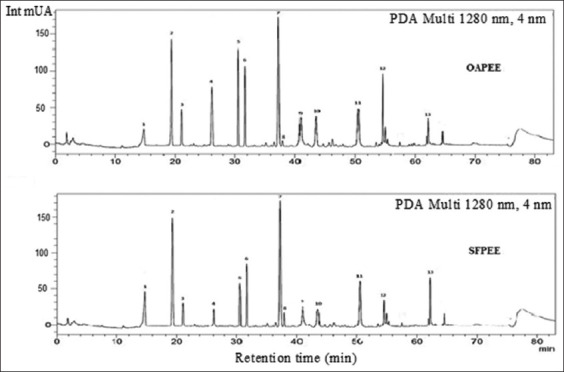
Chromatograms of OAPEE and SFPEE with identified phenolics, (1) vanillic acid, (2) epicatechin, (3) coumaric acid, (4) ferulic acid, (5) ellagic acid, (6) hesperidin, (7) apigenin, (8) cinnamic acid, (9) rosmarinic acid, (10) rutin, (11) naringin, (12) quercetin and (13) kaempferol. OAPEE=Oued Amlil propolis ethanolic extract, SFPEE=Sefrou propolis ethanolic extract.

The quantification of phenolic compounds in the propolis samples was performed using HPLC-DAD. The results revealed the presence of 13 compounds, with the highest concentration registered for epicatechin (310±28.30 mg/g) in the OAPEE and for apigenin (410±21.20 mg/g) in the SFPEE. However, gallic and chlorogenic acids were not detected in either sample (Table-5 and Figure-1). The results obtained here indicated a similarity in phenolic compounds among the propolis samples, regardless of their botanical and geographical origin. Similarly, in a study conducted by Osés *et al*. [52], p-coumaric and ferulic acids were found in 13 propolis samples from different geographical areas.

**Table 5:** Phenolic compounds of Moroccan propolis studied.

Phenolic Acids/Flavonoids	Propolis samples	

	OAPEE (mg/g)	SFPE SFPEE (mg/g)
Vanillic acid	3±0.70	13±2.83***
Epicatechin	310±28.30	269±26.9
Gallic acid	ND	ND
Coumaric acid	6.41±0.58	16±4.24**
Ferulic acid	28.80±3.96**	7.60±0.84
Ellagic acid	98±4.24*	56±7.07
Chlorogenic acid	ND	ND
Hesperidin	101±4.24	145±9.90*
Apigenin	245±21.20	410±21.20***
Cinnamic acid	18±1.41	38±2.12**
Rosmarinic acid	47±2.83	68±4.24**
Rutin	35.9±2.69	60.0±2.83**
Naringin	68±4.24	99±2.83**
Quercetin	87±7.07**	32±2.83
Kaempferol	107±7.07	150±2.83*

OAPEE=Oued Amlil propolis ethanolic extract, SFPEE=Sefrou propolis ethanolic extract. SD=Standard deviation for duplicate determination; the results are presented as mean±SD.

* (p<0.05),

** (p<0.01),

*** (p<0.001)

The phenolic composition of propolis can be used to standardize this beehive product. For instance, Özkök *et al*. [53] showed that caffeic acid, caffeic acid phenethyl ester, galangin, and pinocembrin could be markers of Turkish propolis. In turn, Polish propolis was characterized by *p*-coumaric acid, 2-acetyl-1,3-di-*p*-coumaryl glycerol, and *p*-coumaric acid benzyl ester, together with galangin and chrysin as the main polyphenols [54]. In Italian propolis, pinocembrin was one of the most important flavonoids, and isoferulic, ferulic, and caffeic acids were the major phenolic acids [55]. The antifungal activity of propolis against yeasts is attributed to its rich composition in phenolic acids, flavonoids, and esters [56]. In this study, the ethanolic extracts of the Moroccan propolis samples proved their efficacy *in vitro* against all clinical *Candida* isolates. The obtained MIC and MFC values were close to the reference drug fluconazole (Table-6). These results are superior to those reported for Romanian propolis against *Candida* strains, which ranged between 230 μg/mL and 15000 μg/mL [57]. However, it was found that Turkish propolis was more potent in the inhibition of *C. albicans* and *C. glabrata*, as its MIC values ranged from 0.006 to 0.05 and from 0.025 to 0.1 μg/mL, respectively [58]. In contrast, in Spanish propolis, the antifungal activity against *C. glabrata* was supported by MIC values ranging between 60 and 240 μg/mL [59].

**Table 6:** Anticandidal activity of Moroccan propolis against clinical *Candida* species.

Samples	*Candida* species			

	*Candida albicans*	*Candida glabrata*	*Candida krusei*	*Candida parapsilosis*
OAPEE				
MIC (µg/mL)	62.5	62.5	62.5	31.2
MFC (µg/mL)	125	125	125	62.5
R	2	2	2	2
SFPEE				
MIC (µg/mL)	62.5	62.5	62.5	31.2
MFC (µg/mL)	125	125	125	62.5
R	2	2	2	2
FLZ				
MIC (µg/mL)	46	46	93	37.5
MFC (µg/mL)	93	93	187	75
R	2	2	2	2

OAPEE=Oued Amlil propolis ethanolic extract, SFPEE=Sefrou propolis ethanolic extract, MIC, MFC=Concentrations by µg/mL, FLZ: fluconazole standard drug. R=MIC/MFC, MIC=Minimum inhibitory concentration, MFC=Minimum fungicidal concentration

The anticandidal activity of propolis may be attributed to its polyphenol content. For instance, in a study conducted by Quiroga *et al*. [60], it was found that pinocembrin and galangin isolated from Argentinian propolis were partially responsible for its fungitoxic activity. In addition, vanillin, 4-coumaric acid, and methyl ferulate are among the molecules reported to be good inhibitors of biofilm formation in microorganisms, including *C. albicans* [61].

It was proven that the antifungal activity of propolis can be attributed to the inhibition of the formation of the hyphal forms of C. albicans, which represent an important factor of virulence of these pathogenic yeasts. In addition, it was reported that a mixture of antifungal drugs with propolis can lead to synergistic effects and improved results [62].

## Conclusion

The Moroccan propolis showed good physicochemical characteristics and rich chemical composition, including minerals, flavonoids, and phenolic acids. In addition, they were endowed with an important antifungal activity, which renders them a potential therapeutic agent that may be useful in the prevention and treatment of candidiasis infections. Further studies are needed to identify the bioactive molecule responsible for the antimycotic activity of propolis and to determine its mechanism of action.

### Data Availability

The supplementary data can be available from the corresponding author upon a reasonable request.

## Authors’ Contributions

AA: Propolis harvesting, extraction, antioxidant and antifungal activity, and drafting of the manuscript. ST: Propolis sampling and clinical candida strain isolation and the interpretations of the results. MF: Performed data treatment and physicochemical analysis. BL: Conceived and designed the study. All authors read and approved the final manuscript.
